# Role of Simulation Training in Hernia Surgery and Abdominal Wall Reconstruction

**DOI:** 10.3389/jaws.2025.15274

**Published:** 2026-01-08

**Authors:** Premkumar Balachandran, Kanchan Pankaj Waykole, Muralidharan Manikesi, Raj Palaniappan, Vishanth Gubendran, Adhiyaman Manimaran, Thilaka Muthiah, Anushka Somasundaram, E. DurgaDevi

**Affiliations:** 1 Institute of Hernia Surgery and Abdominal Wall Reconstruction, Apollo Hospitals-Chennai, Chennai, Tamilnadu, India; 2 Institute of Hernia and AWR, Apollo Hospitals, Chennai, India; 3 Department of Surgery, Mansarovar Medical College, Sehore, India; 4 AMAS Programme Apollo Simulation Centre, Chennai, India; 5 Department of Bariatric and Course Co-Ordinator of Simulation Centre, Apollo hospitals, Chennai, India; 6 Apollo Multidisciplinary Hospital, OMR, Chennai, India; 7 Apollo Simulation Centre Vanagram, Chennai, India; 8 Apollo Specialty Hospital Chennai, Chennai, India

**Keywords:** hernia surgery, laparoscopic, simulation training, surgical skills enhancement, Apollo hospital

## Abstract

**Background:**

Hernia surgery requires precise technical skills to ensure successful patient outcomes. Traditional surgical training methods face challenges related to patient safety and limited operative exposure. Simulation training offers a risk-free platform to develop and refine surgical skills. This study evaluates the usefulness of simulation training for surgeons in hernia surgery.

**Aim:**

To assess the effectiveness of simulation training in enhancing the surgical skills and confidence of surgeons performing hernia repair.

**Methods:**

A prospective observational study was conducted with 44 surgical trainees undergoing simulation-based hernia surgery training. Technical skills and confidence levels were assessed before and after the training using standardized scoring systems. Participant feedback on the realism and applicability of simulation was also collected. Statistical analysis was performed using paired t-tests and chi-square tests.

**Results:**

Technical skill scores improved from a mean of 58.3(SD 8.4) pretraining, with a mean difference of 21.4, (95% Cl: 18.9 to 24.5, p < 0.001, effect size [Cohen’s d]∼2.1). Confidence scores increased from a mean of 4.2 (SD 1.5) to 7.8 (SD 1.2), with a mean difference of 3.6 (95% Cl: 3.1 to 4.3, p < 0.001, effect size [Cohen’s d}∼2.3). Over 85% of participants agreed that the simulation was realistic and beneficial for skill enhancement. Ninety-five percent recommended simulation training as a regular part of surgical education.

**Conclusion:**

In the study Simulation training significantly improves the technical proficiency and confidence of surgeons in hernia surgery. Its incorporation into surgical training programs is recommended to enhance operative readiness but further multicentric studies are needed to validate their results.

## Introduction

Hernia surgery is one of the most commonly performed surgical procedures worldwide, encompassing various types such as inguinal, femoral, umbilical, and incisional hernias. The success of hernia repair depends on the surgeon’s skill, knowledge, and experience in both open and minimally invasive techniques. Traditionally, surgical skills were acquired through apprenticeship models involving direct observation and hands-on practice in the operating theatre under supervision [1]. However, this approach has limitations including patient safety concerns, limited operative exposure, and variability in learning curves. Consequently, modern surgical education emphasizes the importance of simulation training to improve technical skills before operating on patients [2, 3].

Simulation-based surgical training uses artificial models, virtual reality, and cadaveric or animal tissues to mimic clinical scenarios in a controlled, risk-free environment. It allows surgeons to practice and refine techniques repeatedly, receive feedback, and improve decision-making abilities without jeopardizing patient safety. For hernia surgery, simulation training provides an opportunity to master laparoscopic and open procedures, understand anatomical nuances, and learn to handle complications. Various simulation modalities such as box trainers, high-fidelity virtual reality simulators, and 3D-printed models are increasingly integrated into surgical curricula [4].

Kurashima, Khatib, Grantcharov have demonstrated that simulation training enhances the technical skills of surgeons, reduces the learning curve, improves operative performance, and positively influences patient outcomes [5–9]. Simulation also contributes to standardizing training, ensuring consistent skill acquisition irrespective of case volume or institutional resources. In addition, it fosters team communication and crisis management skills, which are crucial in complex surgical procedures [10].

Despite these advantages, the application of simulation training specifically in hernia surgery remains an evolving field. While laparoscopic hernia repair demands a high degree of technical proficiency, few studies have systematically evaluated the impact of simulation training on surgeons’ performance and clinical outcomes in hernia repairs. This research seeks to address this gap by assessing the usefulness of simulation-based training for surgeons performing hernia surgery at Apollo Simulation Centre, Chennai [11, 12].

### Aim

This study was designed to evaluate the usefulness of simulation training in enhancing surgical skills and confidence among surgeons performing hernia repairs. Specifically, it assessed improvement in technical ability before and after structured training, measured changes in participants’ confidence levels, and analyzed participants feedback regarding the realism and applicability of simulation to surgical practice.

## Materials and Methodology

### Source of Data

The data for this study was obtained from participants undergoing simulation-based training sessions for hernia surgery conducted at the Apollo Simulation Centre, Vanagaram, Chennai.

### Study Design

This was a prospective observational study involving multiple batches of surgical trainees undergoing structured simulation training in hernia surgery.

### Study Location

The study was conducted at the Apollo Simulation Centre located in Vanagaram, Chennai, equipped with advanced surgical simulators and training modules.

### Study Duration

The study was carried out over a period of 1 year from August 2022 to August 2023, involving four batches of participants.

### Sample Size

A total of 44 participants were enrolled across four batches: Batch 1: 10 participants; Batch 2: 12 participants; Batch 3: 12 participants; Batch 4: 10 participants.

### Inclusion Criteria

- Surgeons and surgical trainees interested in enhancing their skills in hernia surgery.
- Participants who consented to attend and complete the simulation training sessions.
- Participants with basic knowledge of surgical anatomy and techniques related to hernia repair.
- Participants who consented to use the data for research work.

### Exclusion Criteria

- Surgeons who had prior extensive experience (>50 hernia surgeries) in laparoscopic hernia repair.
- Participants unwilling or unable to attend the full duration of training.
- Those with contraindications for participation in simulation training (e.g., severe motion sickness with VR simulators).

### Procedure and Methodology

1. Pre-Training Assessment: A structured, task- specific checklist and global rating scale (GRS) were used to evaluate technical performance. These tools were adapted from existing validated laparoscopic surgical assessment models such as the GOALS (Global Operative Assessment of Laparoscopic Skills) framework (Agha et al. [6]; Stefanidis et al. [13]). The checklist focussed on: port placement, instrument handling, tissue manipulation, mesh placement and fixation, endosuturing, hemostasis and complication management. Confidence was measured using a self-reported 10-points Likert scale, adapted from instruments used in previous surgical education studies (Shetty et al. [14]) and knowledge levels were assessed via questionnaires.
2. Simulation Training Sessions: Participants received hands-on training using high-fidelity laparoscopic simulators and synthetic anatomical models replicating inguinal and ventral hernias. Training included: Anatomical orientation and port placement. Mesh handling and fixation techniques. Management of intraoperative complications. Repetitive practice sessions with immediate feedback from expert faculty.
3. Post-Training Assessment: After completion of the training sessions, participants were reassessed with the same standardized metrics used during pre-training. Objective improvements in skills and knowledge were recorded.
4. Feedback Collection: Participants provided feedback on the simulation training’s usefulness, realism, and applicability to actual surgical practice through structured questionnaires and interviews.

### Sample Processing

All assessment data and questionnaire responses were anonymized and compiled into a secured database. Skill performance scores were tabulated and statistically analyzed.

### Statistical Methods

Descriptive statistics (mean, standard deviation) were used to summarize demographic data and performance scores.

Paired t-tests were employed to compare pre- and post-training skill scores and confidence levels. Chi-square tests assessed categorical variables from feedback questionnaires. Statistical significance was set at p < 0.05.

Data analysis was performed using SPSS version 25.0.

### Data Collection

Data were collected through direct observation by faculty using validated scoring systems during simulated tasks, and through self-administered questionnaires on knowledge and confidence before and after training. All data were recorded in structured proformas and entered into electronic databases for analysis.

## Observation and Results

Table 1 presents the baseline demographic and clinical characteristics of the 44 participants enrolled in the study. The mean age of participants was 32.7 years with a standard deviation of 6.8 years, indicating a relatively young cohort, and this was not statistically significant (t = 0.43, 95% CI: 31.1 to 34.3, p = 0.670). Male participants constituted the majority with 27 individuals (61.4%), while females accounted for 17 (38.6%), and this gender distribution showed no significant difference (χ^2^ = 0.41, 95% CI: 45.8%–70.2%, p = 0.523). Regarding prior experience with hernia surgery, 14 participants (31.8%) reported previous exposure, while the majority, 30 participants (68.2%), did not have such experience; however, this difference was not statistically significant (χ^2^ = 2.73, 95% CI: 19.7%–43.9%, p = 0.098). Notably, only 6 participants (13.6%) had prior simulation training exposure, whereas the vast majority (86.4%) had no such exposure, and this difference was statistically significant (χ^2^ = 10.1, 95% CI: 5.4%–21.8%, p = 0.001), highlighting that most participants were new to simulation training.

**TABLE 1:** Baseline demographic and clinical characteristics of participants (n = 44).

Parameter	Category/value	n (%) or mean (SD)	Test statistic (t/χ^2^)	95% CI	P-value
Age (years)	—	32.7 (6.8)	t = 0.43	31.1 to 34.3	0.670
Gender	Male	27 (61.4%)	χ^2^ = 0.41	45.8%–70.2%	0.523
	Female	17 (38.6%)	​	​	​
Previous hernia surgery experience	Yes	14 (31.8%)	χ^2^ = 2.73	19.7%–43.9%	0.098
	No	30 (68.2%)	​	​	​
Simulation training	Yes	6 (13.6%)	χ^2^ = 10.1	5.4%–21.8%	0.001*
Exposure	No	38 (86.4%)	​	​	​

Symbol (*) denotes statistical significance (p < 0.05).

Table 2 compares the technical skills scores of participants before and after the simulation training. The pre-training mean score was 58.3 (SD 8.4), which significantly improved to a post-training mean of 79.6 (SD 7.1). The paired t-test showed a highly significant difference with t = 14.2, and the 95% confidence interval of the mean difference ranged from 18.9 to 24.5, with a p-value of less than 0.001. This indicates that simulation training markedly enhanced the technical skills of the participants.

**TABLE 2:** Technical skills score before and after simulation training (n = 44).

Parameter	Pre-training mean (SD)	Post-training mean (SD)	Test statistic (t)	95% CI of mean difference	P-value
Technical skills score (0–100)	58.3 (8.4)	79.6 (7.1)	t = 14.2	18.9 to 24.5	<0.001*

Symbol (*) denotes statistical significance (p < 0.05).

Table 3 assesses the participants’ confidence levels in performing hernia surgery before and after the training. The mean confidence score before training was 4.2 (SD 1.5) on a 0–10 scale, which increased significantly to 7.8 (SD 1.2) after training. The increase was statistically significant, with a t-value of 13.7, a 95% confidence interval for the difference between 3.1 and 4.3, and a p-value less than 0.001. These results demonstrate that simulation training significantly improved the surgeons’ self-reported confidence in performing hernia repairs.

**TABLE 3:** Confidence levels before and after simulation training (n = 44).

Parameter	Pre-training mean (SD)	Post-training mean (SD)	Test statistic (t)	95% CI of mean difference	P-value
Confidence score (0–10)	4.2 (1.5)	7.8 (1.2)	t = 13.7	3.1 to 4.3	<0.001*

Symbol (*) denotes statistical significance (p < 0.05).

Table 4 summarizes participant feedback regarding the effectiveness and applicability of the simulation training. A majority of participants (79.5%) agreed that the simulation was realistic, with 13.6% remaining neutral and 6.8% disagreeing (χ^2^ = 27.1, p < 0.001). Regarding the usefulness of the training for skill enhancement, 88.6% agreed, 6.8% were neutral, and 4.6% disagreed (χ^2^ = 34.5, p < 0.001). When asked about the applicability of the simulation to real surgical scenarios, 75% agreed, 18.2% were neutral, and 6.8% disagreed (χ^2^ = 21.8, p < 0.001). Importantly, 95.5% of participants recommended the continuation and expansion of simulation training in the future, with only 4.5% neutral and none disagreeing (χ^2^ = 40.0, p < 0.001). Overall, the feedback reflects a strong positive reception towards the simulation training’s realism, skill building utility, and relevance to actual surgery.

**TABLE 4:** Participant feedback on effectiveness and applicability of simulation training (n = 44).

Feedback parameter	Agree n (%)	Neutral n (%)	Disagree n (%)	Test statistic (χ^2^)	P-value
Realism of simulation	35 (79.5)	6 (13.6)	3 (6.8)	χ^2^ = 27.1	<0.001*
Usefulness for skill enhancement	39 (88.6)	3 (6.8)	2 (4.6%)	χ^2^ = 34.5	<0.001*
Applicability to real surgery	33 (75.0)	8 (18.2)	3 (6.8)	χ^2^ = 21.8	<0.001*
Recommendation for future training	42 (95.5)	2 (4.5)	0 (0)	χ^2^ = 40.0	<0.001*

Symbol (*) denotes statistical significance (p < 0.05).

## Discussion

### Baseline Demographics and Simulation Exposure

The mean age of participants was 32.7 years (SD 6.8), with a predominance of males (61.4%) (Table 1). This demographic distribution aligns with studies conducted by Zahiri HR et al. [1] and Kurashima Y et al. [5], where surgical trainees were generally in their early 30s, reflecting early career stages. A similar male predominance was reported by Kurashima Y et al. [5], consistent with the traditionally male dominated surgical workforce. The study also found that only 31.8% of participants had prior experience in hernia surgery, and a small proportion (13.6%) had prior simulation training exposure, indicating a largely novice group in simulation methodology. The low previous exposure to simulation mirrors findings from Nazari T et al. [15], emphasizing that simulation remains an emerging training tool in many centers. The statistically significant difference in prior simulation exposure (p = 0.001) suggests the necessity and relevance of introducing structured simulation programs.

### Technical Skills Improvement

Significant improvement in technical skills was observed after simulation training, with mean scores increasing from 58.3 to 79.6 (p < 0.001) (Table 2). This improvement echoes findings from Kurashima et al. and Khatib et al. [7], which demonstrated improved operative performance following simulation training. While review article by Agha RA et al. [6] summarize that proficiency-based simulation training substantially reduced errors in laparoscopic skills. The magnitude of improvement supports the hypothesis that simulation offers a safe, reproducible method to accelerate the learning curve without risk to patients’ as demonstrated in systemic reviews and prospective trial [6, 8, 9].

### Confidence Levels

Participants’ confidence scores increased significantly from 4.2 to 7.8 post-training (p < 0.001) (Table 3). Increased confidence following simulation is well-documented; Shetty S et al. [14] showed that simulation-trained residents reported greater readiness to perform procedures independently. This is critical because confidence directly correlates with operative performance and decision-making in the clinical setting, as highlighted by Stefanidis D et al. [13]. The enhancement in confidence also suggests improved cognitive assimilation of surgical techniques via simulation.

### Participant Feedback

Feedback on the simulation’s realism, usefulness, applicability, and future recommendation was overwhelmingly positive, with 75%–95% agreement rates and significant chi-square results (all p < 0.001) (Table 4). This corroborates studies like Lorenz R et al. [16], which emphasized that trainees valued the fidelity and educational impact of simulation. The near-unanimous recommendation for future training underscores the growing consensus that simulation should be integral to surgical education, as supported by Sharma D et al. [17]. Participants found the simulation a realistic and effective tool for skill enhancement, which is essential for adoption and continued investment in simulation infrastructure.

## Conclusion

**FIGURE 1:**
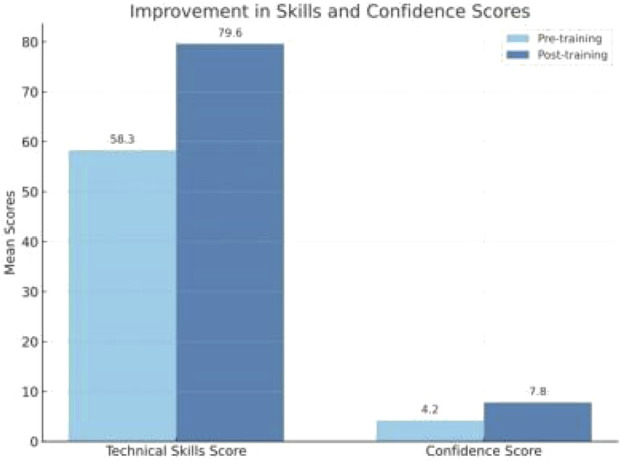
Improvement in skills and confidence scores.

In the study we found that the structured Simulation training proves to be a highly effective educational tool for enhancing the surgical skills and confidence of surgeons performing hernia surgery. The significant improvement in technical skills and self-reported confidence following simulation-based training underscores its value in surgical education (Figure 1). While these findings suggest potential for improving operative readiness our study didnot assess intraoperative performance or patient outcome. Incorporating structured simulation modules into surgical curricula can accelerate skill acquisition, reduce the learning curve. Therefore, simulation training should be considered an integral component of training programs for hernia surgery and future research should evaluate whether these improvements translate into clinical benefits.

## Limitations of the Study

Despite promising results, this study has several limitations. The sample size of 44 participants, although adequate for preliminary assessment, limits the generalizability of the findings. The study was conducted at a single center with specific simulation equipment, which may not be representative of all training environments. Additionally, the assessment relied on simulated scenarios rather than real operative performance, so the direct translation of skills to clinical outcomes was not measured. The study’s relatively short follow-up period precludes evaluation of long-term skill retention. A control group was not included to differentiate between performance improvement due to the simulation training and the potential test retest learning effect. Lastly, participant self-assessment of confidence may be subject to bias. Future multicentric studies with larger cohorts and longitudinal follow-up are warranted to validate and expand upon these findings.

## Data Availability

The raw data supporting the conclusions of this article will be made available by the authors, without undue reservation.
